# Influence of Post-Weld Heat Treatment on Microstructure and Toughness Properties of 13MnNiMoR High Strength Low Alloy Steel Weld Joint

**DOI:** 10.3390/ma14185336

**Published:** 2021-09-16

**Authors:** Songya Tian, Fan Xu, Genyuan Zhang, Adnan Saifan, Bassiouny Saleh, Xiaobo Li

**Affiliations:** 1 College of Mechanical and Electrical Engineering, Hohai University, Changzhou 213022, China; 15851962696@163.com (F.X.); jidyjs1935@sina.com (G.Z.); 141309020005@hhu.edu.cn (X.L.); 2 Mechanical Engineering Department, Faculty of Engineering, Sana’a University, Sana’a 12544, Yemen; 3 College of Mechanics and Materials, Hohai University, Nanjing 211100, China; bassiouny.saleh@alexu.edu.eg; 4 Production Engineering Department, Alexandria University, Alexandria 21544, Egypt

**Keywords:** post-weld heat treatment, 13MnNiMoR weld metal, martensite-austenite constituent, tempering, intercritical normalizing, impact toughness

## Abstract

Weld and base metals require hot or cold working during the steel equipment manufacturing process. As a result, the components should be subjected to a normalizing heat treatment in order to recover their mechanical properties. In this study, the submerged-arc welding of the high strength low alloy (HSLA) thick steel plate(13MnNiMoR) is adapted for the vessel head under the normalizing and tempering heat treatment. The findings showed that the material toughness decreases after heating to simulate a vessel head forming process. The stamping process is carried out under the conditions of 980 °C for one hour, normalizing at 920 °C for 1 h and tempering between 600–660 °C for 2 h, respectively. The martensite-austenite (M-A) constituent is distributed in granular bainite and the boundary of austenite in island constituent. Therefore, it was deemed to be the most detrimental to Charpy-V impact toughness. Between normalizing and tempering, intercritical normalizing at 740 °C was added. As a result of the ferrite with fine particles M-A constituent, the toughness increases significantly.

## 1. Introduction

Welded components typically require cold working or hot forging of steel plates to achieve a specific geometry [1]. A high level of residual stresses is generated during the welding and cold working process of the thick plate, and then heat treatment is performed to relieve such stress [2,3,4]. Because this is always done at temperatures between 600 °C and 700 °C below Ac1, the microstructure and mechanical properties of weld metals may not be significantly altered [5]. In some cases, the thick plates are usually treated by a normalizing and tempering treatment after the cold working and hot forging processes to recover their mechanical properties [6,7]. The normalizing heat treatment temperature for C-Mn and Mn-Si steel welded by submerged-arc welding is up to 920 °C, and the yield strength decreases, while the Charpy V-Notch Impact energy of weld metal increases after normalizing [8,9,10]. However, the microstructure of low alloy high strength steel weld metal is a mixture of two or more constituents, such as proeutectoid ferrite, polygonal ferrite, aligned and non-aligned side plate ferrite, ferrite-carbide aggregate, and acicular ferrite [11,12] or upper and lower bainite, martensite. Therefore, the austenite -martensite (M-A) constituent might be formed.

The complete austenitization of the weld metal imposed by the normalizing heat treatment, associated with its significantly lower thermal cycles compared with the thermal welding cycles, affects the weld joint microstructure [13,14]. The investigation on low alloy steel plate (C:0.04%, Si: 0.04%, Mn:1.1–1.5%, Mo: 0.5%, Ni: 2.6%) by submerged-arc welding with a normalizing treatment of 920 °C was carried out [15,16,17]. It demonstrates that after normalization, the deposited metal had improved tensile strength and toughness. However, the steel plate is only 16 mm thick and has a low carbon content.

Pressure vessels can be used in harsh environments with high temperatures and pressures, as well as extremely low temperatures [18]. Thus, high-quality properties of thick plates are required, including a good combination of strength and toughness; as a result, the quality of welding joints must be improved [19,20]. Moreover, for high tensile strength, the carbon content of the weld metal should be increased [21]. Furthermore, it produces(requires) a high pre-heated temperature and interposes temperature to prevent cold cracks. It is unclear how heat treatment affects welding joints with higher carbon content. As a result, the effects of carbide precipitation on the microstructure and mechanical properties of SA508GR4N steel following tempering and PWHT must be investigated [22,23,24].

The 13MnNiMoR thick plates are widely used in the manufacture of pressure vessels. The high-temperature properties of 13MnNiMoR are studied, and the results show that the granular bainite, ferrite and martensite are in the steel microstructure of the specimen after the hot rolled and normalizing. In addition, some granular bainite is decomposed after tempering, and the tensile and yield strengths are decreased while the specimen ductility is increasing a little bit in the range of 200 °C to 400 °C, and therefore the results meet the standard requirements [25,26].

However, there are few studies on the low-temperature performance of submerged-arc welding joints of this kind of thick steel plate. Some studies discussed the thermal simulation of the coarse zone in the heat-affected zone (HAZ). In that study, the authors indicated that once the peak temperature of the second thermal cycle exceeds 750 °C, the toughness decreases due to a large fraction of martensite-austenite (M-A) constituents. However, the impact toughness is greatly improved during the tempering process at 580–640 °C [27].

Although the mechanical properties of steel have been studied and improved at high temperatures, pressure vessels need to enhance their mechanical properties at low temperatures. The effect of the multi-pass thermal cycle on the impact toughness at 0 °C was also discussed. However, few studies have been conducted to investigate the effect of post-weld heat treatment on welded steel components, which is governed by CN-NB/T 47015 [28]. Thus, the mechanical property of welded components of the steel needs to meet the requirement after heat treatment.

In addition, the thick welded steel plate is constructed as a pressure vessel head, and the H10Mn2NiMoA welding wire matched with SJ102 flux is chosen for submerged-arc welding. When the welding joint obtained in this manner is subjected to hot forging (980 °C), normalizing (920 °C), and tempering (600–660 °C), the toughness decreases dramatically [28]. A 740 °C intercritical normalizing is added between normalizing at 920 °C and tempering between 600–660 °C to increase the toughness of the welding joint at −20 °C [29]. In order to further improve the applicability of 13MnNiMoR HSLA steel weld in pressure vessel production, it is essential to understand the relationship between impact toughness and weld metal microstructure. Therefore, the purpose of the study is to investigate the microstructure and toughness of weld metal after post-welding heat treatment.

## 2. Experimental Procedures

### 2.1. Materials and Sample Preparation

13MnNiMoR steel has high strength, which is suitable for the high-pressure vessel, shielded metal arc welding is used for backing weld while submerged-arc welding is used to fill the groove of the welding joint with high efficiency.

In this work, the experiment base dimension is 150 mm × 900 mm × 60 mm. The groove in the x-axis has a root gap and root height of 2 mm, with an angle of 60°. Table 1 shows the chemical composition and mechanical properties of the base metal that is used in this study. The backing weld is manually achieved using the E6015 electrode, and the rest of the weld is done via submerged-arc welding. The chemical composition of welding flux and welding wire is shown in Table 2 and Table 3, respectively. Taking into account the stable welding process and the microstructure of the welding joint, Table 4 shows the welding parameters of the experiment. The Preheat and inter-pass temperatures are controlled in the range of 220–230 °C.

### 2.2. Post-Weld Heat Treatment Conditions

The post-welding heat treatment process was performed as follows: the weld joint was heated to 980 °C and held for 2 h to simulate the hot stamping forming process, and A specimen with 75 mm length and a specimen with 5 mm length was cut from the welded plate normalized at 980 °C along the direction of the weld to make the impact specimens of three welds and three heat-affected zones, one specimen for observation microstructure.

The rest of the welded plate was heated to a normalizing temperature of 920 °C for 1 h, and similarly, a welding joint with 75 mm length is cut off for 6 pieces of impact specimens for HAZ and weld at 920 °C normalizing. Likewise, a welding joint with 5 mm length is cut off to observe the microstructure.

Lastly, the rest of the welded plate was cut into two pieces; one part is 260 mm in length, it is divided into three blocks with equal length, they are tempered for 2 h, 620 °C, 640 °C, and 660 °C, respectively. In addition, the total number of samples without intercritical normalizing at 740 °C is 18 for impact testing, and three specimens were selected for microstructure observations.

The other part, 340 mm length, is normalized at 740 °C for 1 h, called the intercritical normalizing. Welding joint with 75 mm length is taken for impacted specimens of 3 pieces of weld and 3 pieces of HAZ from intercritical normalizing welded plate. In addition, a welding joint with 5 mm length is for microstructure.

Finally, the rest of the welding joints are cut into three pieces, each specimen length is 80 mm, and then tempered at 620 °C, 640 °C, and 660 °C, for 2 h, respectively. They are machined into 6 pieces of specimens and a piece of microstructure specimens, respectively.

### 2.3. Characterization Methods

In this study, the electrical discharge cutting machine has been used to cut impact samples. Based on CIEM-30D CPC E23 standard [30,31], Charpy impact tests were performed on Charpy V notch specimens (size: 10 mm × 10 mm × 55 mm) of the weld and HAZ at −20 °C. To avoid the influence of the V-notch placement, the Charpy V-notch is perpendicular to the welding layer. The total absorbed energy was determined to represent the impact toughness.

The specimens with dimensions of 20 mm × 20 mm × 4 mm were etched with 4% nital for 12 s for exhibiting the microstructure of weld and HAZ. Lepera’s reagent (4 g of picric acid in 100 mL Ethyl alcohol and 1.5 g Sodium metabisulphite, Na_2_S_2_O_5_ in 100 mL distilled water) is used to clearly reveal the morphologies of MA constituents [32]. For better etching, first etching with 2–3% nital solution for 7 s and subsequently Lepera’s reagent for 14 s. After etching with the Lepera’s reagent, the MA constituents appear white, and the matrix appears dark grey by Olympus BX51Moptical microscopy (Olympus BX51M, Shinjuku, Tokyo, Japan). An energy dispersive X-ray spectrometer (EDX) EX-54175JMU that was made in Japan is used for detecting the compositions in the microzone.

The area percentage of the M-A constituents was counted by Image-ProPlus 6.0 software based on the grayscale among the microstructures, and the appropriate gray level is selected as the threshold. In addition, the M-A constituent area on the metallographic photo was colored red. Additionally, the difference zone between the colored area (the actual M-A constituent area) and the matrix is constructed, and the inconsistent areas with the M-A constituent zone in the original photo are removed or supplied, as results in the area ratio of the colored area, namely, the proportion of M-A component is calculated.

A threshold is set by the Image-Pro Plus 6.0 software to eliminate the small area of M-A constituents on the metallographic photos. The error is corrected by coloring comparison. The colored metallographic images are zoomed in on with the computer and printed; the dimensions of M-A components were counted by transparent mesh line by line. The mesh dimensionis1 μm × 1 μm in reality.

The rapid quenching expansion tester (L78RITA, Linseis Messgeraete GmbH, Bavaria, BY., Germany) was used to analyze the effect of the austenitizing temperature on the initial and final points of bainite from super-cooled austenite in continuous cooling transformation. The specimens were heated to 720 °C, 760 °C, 800 °C, 810 °C, 820 °C, 840 °C, 870 °C, and 910 °C at the 0.05 K/s (approximate equilibrium phase transition by slow heating), held for 30 min and cooled to the room temperature at a rate of 2.5 K/s, respectively, and thus the initial and final temperature of granular bainite transformation at different austenitizing temperatures is calculated by the tangent method.

## 3. Results

### 3.1. Microstructure Analysis

**Figure 1:**
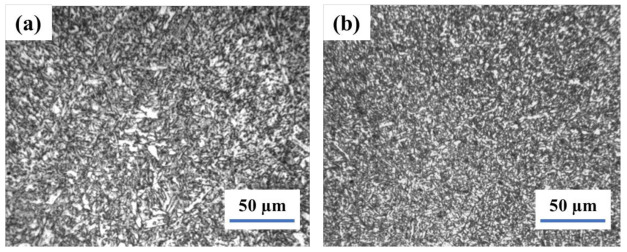
The microstructure of welding joint in as weld−weld (**a**) and HAZ (**b**).

The results of weld and heat-affected zone (HAZ) metallographic analysis in as-welded conditions are illustrated in Figure 1. The weld involves acicular ferrite and fine polygonal ferrite, while the HAZ contains the fine bainite and ferrite, which is good for impact toughness. Furthermore, once the subsequent layer of the weld joint applies heat treatment to the former layer, the columnar crystal in the weld zone is indistinct.

**Figure 2:**
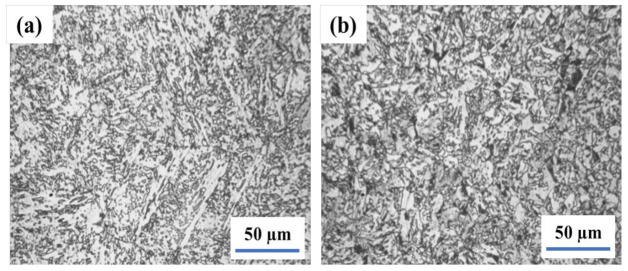
The Microstructure of weld joint after simulation hot stamping−weld (**a**) and HAZ (**b**).

Figure 2 illustrates the weld and heat-affected zone microstructure of a submerged-arc welding joint heated at 980 °C for 2 h and stimulated via the hot stamping forming process. The coarse granular bainite in the range of 50–80 μm can be seen in the weld zone and HAZ [33,34]. The island M-A constituents are scattered in granular bainite. It might be led to a higher tensile strength of the weld joint with low toughness.

**Figure 3:**
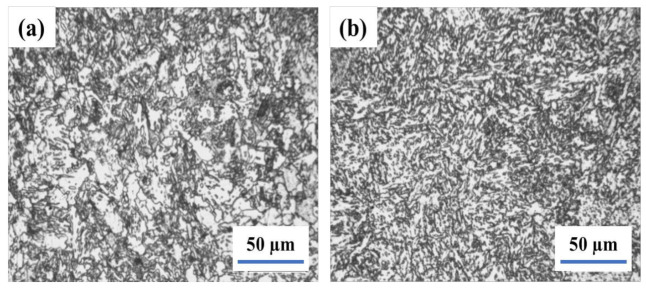
The Microstructure of weld joint after normalizing at 920 °C−weld (**a**) and HAZ (**b**).

The microstructure of the welding joint, which was normalized at 920 °C for 1 h after the hot stamping forming simulation, is depicted in Figure 3. After that, the bainite ferrite in the weld became equiaxial. In the HAZ, the shape of bainite ferrite was transformed into the strip. However, the morphology of the M-A constituents is not significantly changed.

**Figure 4:**
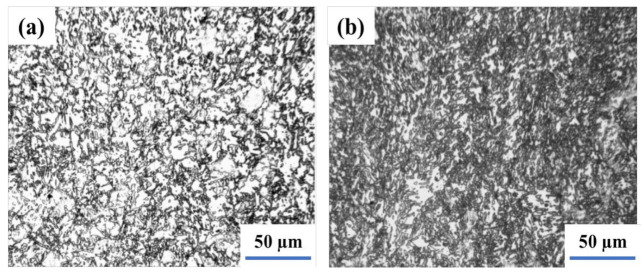
The Microstructure of weld joint after intercritical normalizing at 740 °C−weld (**a**) and HAZ (**b**).

Therefore, the welding joint is treated as intercritical normalizing at 740 °C after being normalized at 920 °C for 1 h and the microstructure is illustrated in Figure 4. Moreover, the Ac1 and Ac3 temperatures of 13MnNiMoR are 709 °C and 821 °C, respectively. During the heating process, the welding joint is in the F + γ region. The austenite is converted into granular bainite, and ferrite is not changed after cooling [35]. Thus, the bainite ferrite that exited in the weld and HAZ became small.

**Figure 5:**
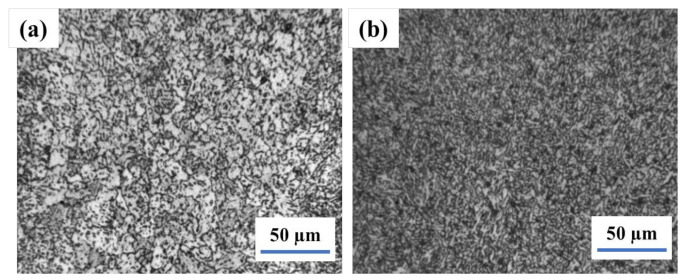
The Microstructure of weld joint after tempering at 660 °C followed by intercritical normalizing−weld (**a**) and HAZ (**b**).

The weld joint was normalized by the intercritical normalizing whose temperature is between Ac1 and Ac3, and the original austenite grain is divided into several parts by ferrite. However, the alloy elements are not easy to diffuse along with the long-distance. Moreover, the dispersed M-A islands that have less carbon and alloy elements are precipitated and distributed in the fine bainite ferrite. Due to the discomposing of particle M-A into the ferrite and carbide during tempering at 660 °C, the M-A constituent can be changed into ferrite and carbide clusters [36]. After intercritical normalizing at 740 °C, the welding joint is tempered at 660 °C for 2 h. The weld and heat-affected zone microstructure are shown in Figure 5. The M-A constituents in the weld were decomposed into black points. It is generally considered that during the tempering process, the segregation of carbon and the precipitation of carbide would appear in bainite ferrite. Besides, the reversion and re-crystallization of ferrite in the weld have appeared as well. Moreover, the granular bainite in HAZ was changed into fine and carbide and thus, the toughness of the welding joint is completely improved.

### 3.2. Martensite-Austenite Constituent Analysis

**Figure 6:**
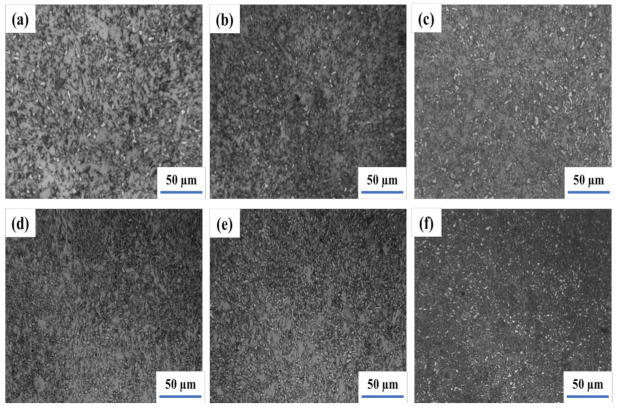
The M−A of the weld joint after hot stamping simulation at 980 °C, normalizing at 920 °C, intercritical normalizing at 740 °C; Simulation−weld (**a**)and HAZ (**b**); normalizing−weld (**c**) and HAZ (**d**); intercritical normalizing−weld (**e**) and HAZ (**f**).

The M-A constituent of submerged-arc welding joint during the hot stamp formation simulation at 980 °C for 2 h, normalizing at 920 °C for 1 h, and intercritical normalizing at 740 °C for 1 h is illustrated in Figure 6. Table 5 explains the M-A constituent size and density after each of the above-mentioned heat-treatment processes. After hot stamping simulation at 980 °C for 2 h, the morphology of the M-A constituent located in the boundary and interior of grain is slender and block shape in the weld and HAZ. Consequently, the M-A constituent amount is 11.43% in the weld and 8.84% in the HAZ. Furthermore, the size of the M-A constituent is 6 μm and 4 μm in the weld and HAZ, respectively. Moreover, after normalizing at 920 °C for 1 h, the size of M-A constituent in weld increases to 11 μm and decreases to 2.5 μm in the HAZ. The destiny of M-A constituent in the weld and HAZ increased to 19.69% and 20.52%, respectively. The M-A constituent size of the welding joint that was heated at 740 °C for 1 h (intercritical normalizing) after normalizing is 4 μm in the weld, and 2.2 μm in the HAZ, while the amount of M-A constituent particles increases in the weld and HAZ. the effect of the shape and distribution of the M-A constituent on the impact toughness is discussed; however, the effect of the size and quantity of the M-A constituent on the impact toughness is not discussed [37].

**Figure 7:**
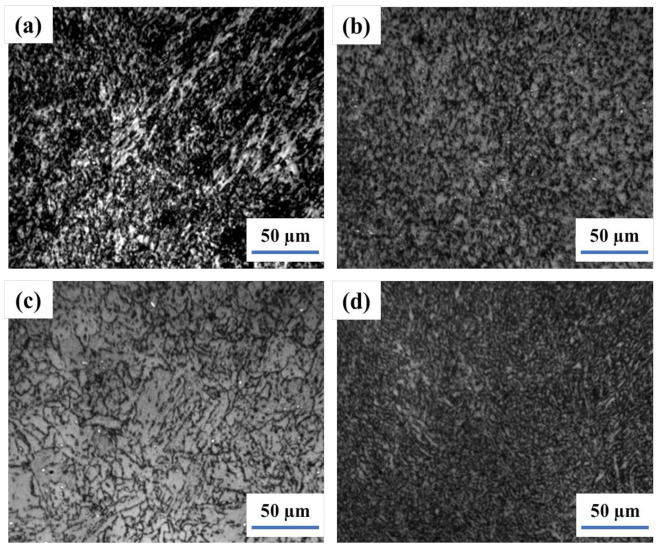
The M−A of the weld joint tempered at 620 °C, at 660 °C after IN: 620 °C−weld (**a**) and HAZ (**b**); 660 °C−weld (**c**) and HAZ (**d**).

Figure 7 shows the morphologies of the M-A constituent of joint tempered at 620 °C and 660 °C for 2 h after intercritical normalizing, respectively. The M-A constituent in the weld and HAZ is a dot round shape located in the boundary and interior of grain. The microstructure of the welding joint tempered at 620 °C for 2 h after intercritical normalizing remains (morphologies of bainite ferrite from normalizing partly), and the amount of M-A constituent in the weld and the HAZ was 1.89% and 2.41%, respectively, less than the amount of intercritical normalizing. Consequently, the size of the M-A constituent is 1.7 μm in the weld and 2 μm in the HAZ, which is less than that of intercritical normalizing.

The welding joint is tempered at 640 °C for 2 h after intercritical normalizing, and the amount of M-A constituent in the weld was 1.21%, less than that after tempering at 620 °C, and decreased to 2.13% in the HAZ. Besides, the size of the M-A constituent in the HAZ was 1.8 μm, while in the weld it was almost kept unchanged compared with that after tempering at 620 °C (as shown in Table 6). In addition, the amounts of M-A constituent in the weld and HAZ tempered at 660 °C for 2 h after intercritical normalizing is lower than that after 640 °C tempering, with 0.83% and 0.43%, respectively. Similarly, the sizes of M-A constituents in the weld and HAZ are also decreased to 1.3 μm and 1.2 μm, respectively. Quenching and intercritical normalizing pre-tempering and tempering is adopted for M-A constituent decomposition into ferrite and small carbides, and reduction of microcrack nucleation sites, but the weld components cannot be quenched for the sake of safety, so the method is difficult to use in the field [38].

### 3.3. Impact Test Evaluation

The impact toughness of the weld joint at −20 °C for as-welded and different heat treatments was listed in Table 7. The toughness of the heat-affected zone and weld in as-welded met the national standard requirements. The difference between the values of weld toughness energy (59 J/cm^2^ and 49 J/cm^2^) results from the position of Charpy specimen notch being located at different zone (original weld and coarse zone reheated by subsequent weld pass).

After a welding joint is normalized at 980 °C to simulate hot stamping forming, normalized at 920 °C and intercritical normalized at 740 °C, the toughness in weld and HAZ is reduced in the range of 8–19 J/cm^2^. Additionally, the welding joint is then tempered after intercritical normalizing, and the impact toughness of the weld and heat-affected zone increases significantly. The impact toughness of weld tempered at 620 °C and 640 °C are similar, which are much lower compared with that of HAZ. While as the welding joint tempered at 660 °C, the impact toughness of weld and HAZ are higher than the one of national standard requirements [39], which could meet the requirement of industrial production, the result is similar to that of Qin Jian’s experiment [40]. The research about the effect of M-A constituent size, density, morphology on impact toughness is turned from qualitative to quantitative [41].

**Figure 8:**
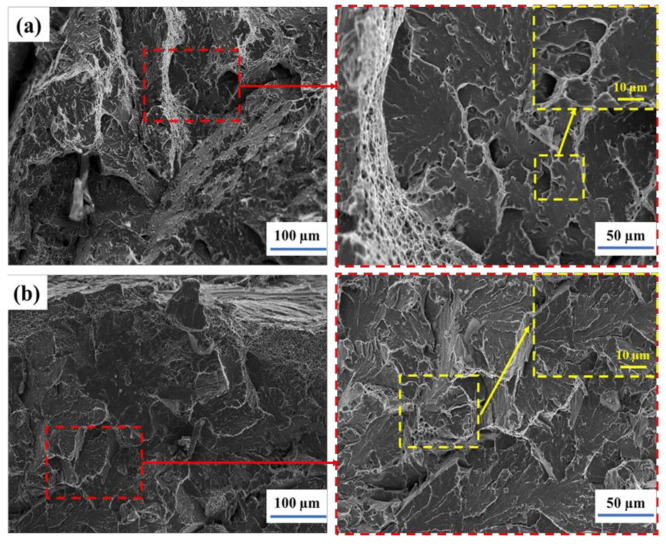
Fracture morphology of weld (**a**) in temper at 660 °C after intercritical normalizing and (**b**) in normalizing after stamp forming.

In this study, only ductile dimple fracture and quasi-cleavage fracture were observed in the surface of all specimens welded and treated by stamp forming at 980 °C, normalizing at 920 °C, intercritical normalizing at 740 °C, and tempering at 660 °C. Figure 8a shows the fracture morphology of weld in a temper at 660 °C after intercritical normalizing. As shown in Figure 8b, the fracture morphology of the weld specimen in normalizing after stamp forming is quasi-cleavage fracture or cleavage fracture.

Table 7 shows the calculated coefficient of variation of impact toughness. The coefficient of variation ranges from 2.9% to 16.3%, with the value being low due to the heat treatment specimen. Nonetheless, the welding joint was welded for multi-layers and multi-beads, the microstructure of the weld and HAZ is not uniform throughout, and the microstructure heredity phenomenon cannot be completely eliminated after heat treatment. As a result, the impact toughness of the weld and the HAZ differ little.

### 3.4. The Stress Field Calculation

**Figure 9:**
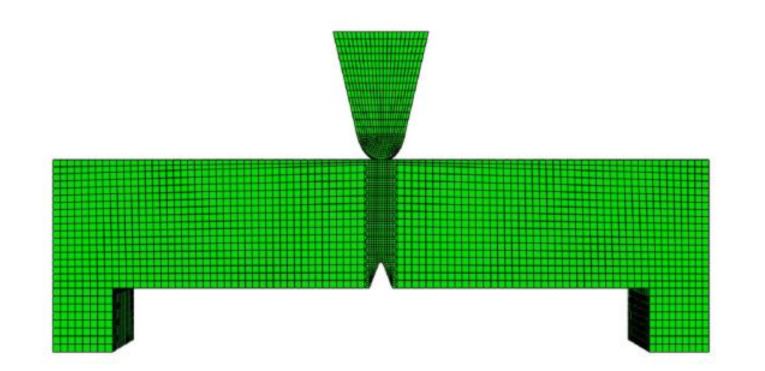
Mesh diagram of Charpy V−shaped impact specimen.

ABAQUS version 2017 software (Dassault Systemes Simulia Corp., Johnston, RI, USA) was used to calculate the stress field at the root of the v-notch in the three-point bending process. ABAQUS software calculates the normal stress σ_yy_ distribution near the root of the V-notch of the Charpy impact specimen using the 8-node plane strain reduced integration element three-dimensional model (C3D8R). The total number of nodes is 857, 470, and the total number of meshes is 880, 331. Figure 9 depicts the mesh diagram. The tensile test under static load determines the relationship between stress and strain.

**Figure 10:**
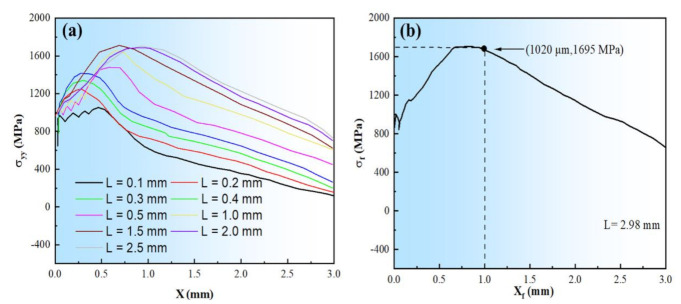
(**a**) The stress distribution at the central axis of v−notch front under different flexible L of three-point bending head and (**b**) the fracture stress at deflection amount of 2.98 mm.

As is well-known, as the deflection amount L and the distance X between peak stress and notch root increase, the peak stress of σ_yy_ on the central axis of the notch front gradually increases. When L reaches a value, the peak stress is no longer significantly increased, but the peak stress with the stress curve becomes wide. Figure 10a demonstrates that σ_yy_ on the central axis of the v-notch front under varying L of the three-point bending head force is distributed. Figure 10b depicts the stress distribution at the notch central plane under different deflections based on the distance between the notch and the source of the crack.

## 4. Discussion

During the impact toughness test, the impact load can be changed at each stage of the deformation and fracture process when it reaches maximum load value Fm. The crack initiation energy Wi includes the elastic deformation work (We) of notch root and plastic deformation work (Wd). Wi reflects the energy dissipation of the crack initiation, determined by the notch root stress concentration and surface state.

**Figure 11:**
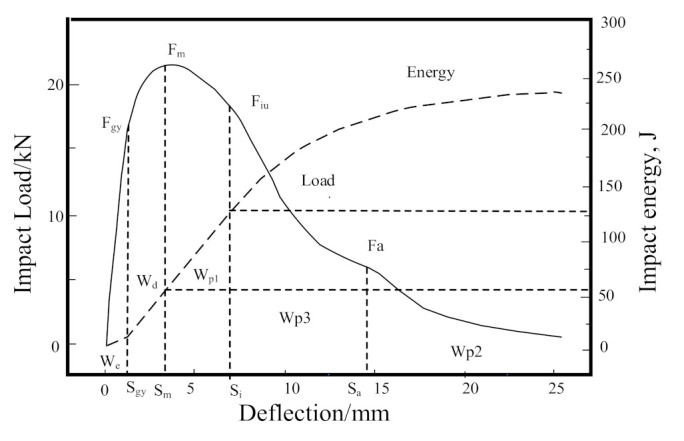
Load-deflection (energy) curves of instrumented impact.

After the crack initiation, the impact sample enters the stage of stable propagation, and the load value decreases slowly with the increase in deflection. When the sample deformation reaches a certain deflection value (the crack reaches the critical length), the load value drops sharply, and the crack expands unsteadily, and then the fracture could be started, as illustrated in Figure 11. The energy consumed by crack steady propagation WP1, unsteady propagation WP3, and the remaining crack propagation energy WP2 is collectively referred to as the crack propagation energy. The total impact energy consumption is mainly determined by crack initiation Wi, steady propagationWP1 and unsteady propagations WP3 [38].

**Figure 12:**
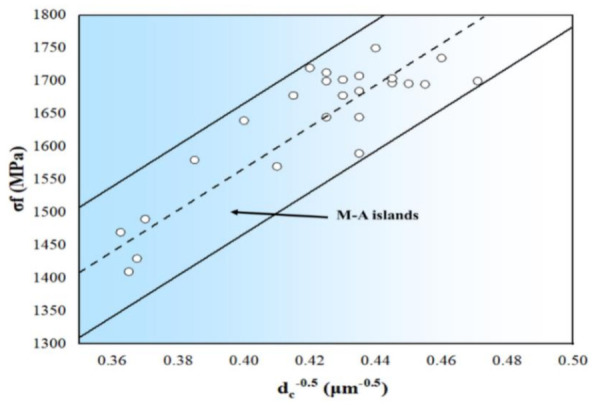
Local fracture stress $\sigma_{f}$ as a function of M−A constituent for three-point bend test samples.

Once the steady propagation energy increases, the crack propagation speed relatively decreases, and therefore the toughness of the material would be better. The impact toughness mainly depends on the crack’s steady propagation energy. Based on Griffith’s crack propagation criterion that was developed by Orowan [42], as the grain or the second phase at the cleavage initiation could induce the micro-crack unsteady propagation, the cleavage fracture stress $\sigma_{f}$ and the critical event size, $d_{c}$, of the grain or the second phase at the crack initiation, must meet Equation (1). In other words, the critical stress, $\sigma_{f}$, of local cleavage fracture is inversely proportional to the square root of the structural parameters (such as inclusions, the second phase, and grain diameter) that induce the cleavage fracture.

(1) $$\sigma_{f}={\left[\frac{\pi E\gamma_{p}}{(1-\upsilon^{2})d_{c}}\right]}^{\frac{1}{2}}$$

where *E* is Young’s modulus and *ν* is Poisson’s ratio of the low alloy steel, which is calculated as 210 GPa and $\gamma_{p}$ is 0.33, respectively. In addition, $d_{c}$ is the length of nonmetallic inclusions, second-phase particles, ferrite grains, or bainite lath, while $\gamma_{p}$ is the effective surface energy of micro-crack propagation. Figure 12 shows the local fracture stress $\sigma_{f}$ for three-point bending specimens as a function of cleavage initiate crack source size.

The $\sigma_{f}$ can be calculated by the distance from the source of cleavage crack to the notch root, and the stress field at the central plane of the V notch from finite element analysis of specimens. The local cleavage fracture $\sigma_{f}$ is inversely proportional to the square root of the M-A constituent diameter, $d_{c}{}^{0.5}$, and the critical event of fracture is mainly related to the M-A constituent length [43,44]. Therefore, after the crack initiation, once the M-A constituent size is closed to the critical event length, the unsteady propagation starts, and the toughness is low. On the other hand, once the M-A constituent length is less than the critical event length after the crack initiates, the crack steady propagation would start and the toughness would be very high. Moreover, after the welding joint is normalized at 980 °C, normalized at 920 °C and intercritical normalized at 740 °C, the M-A constituent length is more than 2 μm, and the toughness in the weld and HAZ is low in the range of 8–19 J/cm^2^. Similarly, after the welding joint being tempered at 620 °C, 640 °C, and 660 °C after intercritical normalized, the M-A constituent length is less than 2 μm, and the toughness in the weld and HAZ is high between 46 and 169 J/cm^2^.

**Figure 13:**
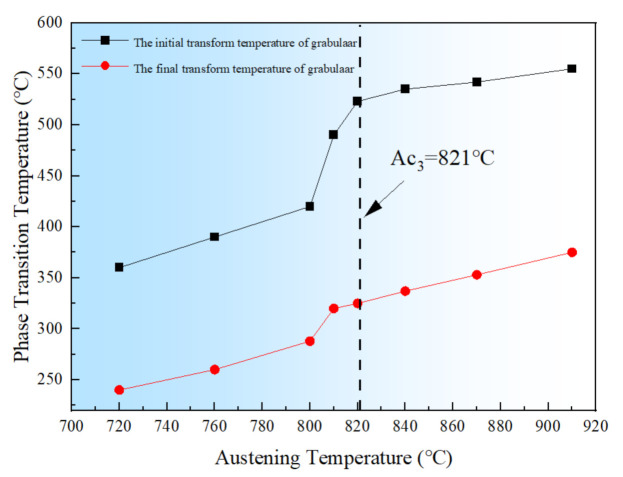
Influence of austenitizing temperature on the initial and final transition temperature of bainite transformation.

It can be seen that from Figure 13, with the decrease in austenitizing temperature, the initial and final transition temperatures of bainite described as B_S_ and B_f_, respectively, are gradually decreased to a certain degree. Furthermore, it is easy to find out that when the austenitizing temperature is lower than 821 °C, as shown in Figure 11, the initial and final transition temperatures of granular bainite decrease more significantly with the decreasing of austenite temperature. Moreover, the decrease in austenitizing temperature and a small amount of undissolved ferrite can increase the stability of super-cooled austenite. It is generally noticed that, as the forming temperature of granular bainite is low, the M-A constituent distributed in the matrix is fine, which can refine the effective grains, and thus the impact toughness of the material is improved.

**Figure 14:**
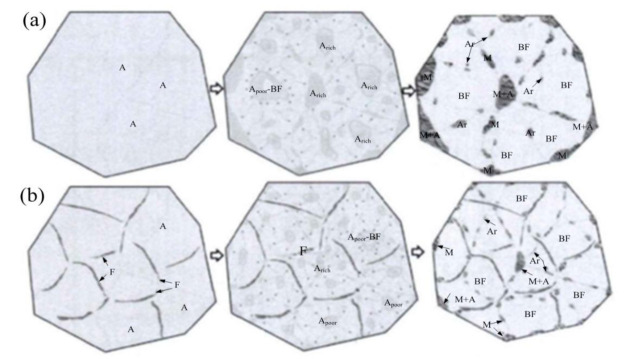
The schematic diagram showing the formation of M−A constituent during granular bainitic transformation (**a**) traditional normalizing process; (**b**) ferrite films formed in the intercritical normalizing process (Where, A: austenite, Arich: rich in composition of C Ni Mn, Apoor: poor in composition of C Ni Mn, BF: bainite ferrite, Ar: retained austenite, M martensite, F: ferrite, and M+A: martensite and austenite).

When the welding joint is normalized at 740 °C, the micrograph is both of F + γ. Therefore, the austenite is converted into granular bainite, but the ferrite is not changed during the cooling process. The thin film of undissolved ferrite can be set in the super-cooled austenite and the grains of super-cooled austenite can be divided effectively, as shown in Figure 14. When the super-cooled austenite is transferred into the granular bainite, the length of carbon diffusion can be reduced, which is the disadvantage to form a large M-A constituent block (especially the large M-A constituent block at the original austenite grain boundary) [45]. Therefore, it is proposed that the small amount of dispersed-distributed undissolved ferrite film can localize the carbon diffusion during the transition of super-cooled austenite into granular bainite, and thus the aim of reducing the large M-A constituent block is ultimately achieved

As the tempering temperature is 620 °C, the obtained ferrite matrix of the granular bainite still retains the same as the one obtained from the normalizing process; therefore, many M-A constituents are distributed in the ferrite matrix. Moreover, with increasing the tempering temperature, the ferrite boundaries tend to be straight, and the granular bainite structure is gradually vanishing. The M-A constituents were decomposed into ferrite and flake carbides patches on the ferrite matrix, and then the carbide is gathered, grew up, and spheroidized. Similarly, when the tempering temperature rises to 640 °C, the ferrite matrix becomes isometric and reverted. Additionally, once the tempering temperature rises to 660 °C, the ferrite matrix has recovery softening and re-crystallization, and the fine grains would appear. Thus, the crack stable propagation WP1 could be increased. The toughness of weld and HAZ is low after stamping simulation at 980 °C due to the coarse granular bainite and massive M-A constituent. Consequently, after normalizing at 920 °C, the toughness is almost kept unchanged while the amount of MA constituent was increased significantly. Meanwhile, the microstructure of granular bainite ferrite in HAZ and weld became slender and blocks, respectively. Furthermore, after tempering at 600 °C, 620 °C, 640 °C and 660 °C followed normalizing at 920 °C, the bainite ferrite in the microstructure did not change. Most of the M-A constituents were decomposed, but the cluster of ferrite and cementite obtained from M-A constituents kept with the original size.

After intercritical normalizing at 740 °C, due to the amount of M-A constituent that is more than that from normalizing at 980 °C and 920 °C, the toughness is still low. During the heat process of intercritical normalizing, some of the bainite ferrites are converted to ferrite, while the M-A constituent with the surrounding bainite ferrite is converted into austenite owing to the high carbon content in the M-A constituent. During the cooling process, the super-cooled austenite is divided into granular bainite and M-A constituent, while the bainite ferrite is segmented by ferrite. Therefore, the amount of new M-A constituent is increased, and M-A constituent length is decreased.

After the tempering process followed by intercritical normalization, the impact toughness of the weld and HAZ increases significantly because many M-A constituents have vanished, and the rest is formed spherically, individually, and fined. After the normalization process at 920 °C, the concentration of Ni and Mn in M-A constituents is 1.18%wt and 1.56%wt, respectively. The result is similar to [46], while 0.83%wt and 0.98%wt after intercritical normalizing at 740 °C. Thus, the M-A constituents from intercritical normalizing are decomposed easily during tempering. The ferrite and austenite both existed during the heat process of normalizing at 740 °C. The concentration of Mo is high in ferrite and low in austenite. After the cooling process, the austenite is converted into granular bainite, whose Mo concentration is less than that of granular bainite after normalizing at 920 °C. The concentration of Mo in ferrite of granular bainite after normalizing at 920 °C is 0.73%wt. Besides, the Mo concentration in equivalent ferrite after intercritical normalizing at 740 °C is 1.03%wt which is obtained by the EDS (Energy Dispersive Spectrometer) method. Consequently, the stability of bainite ferrite from austenite with low Mo concentration is decreased. Moreover, the bainite ferrite could be recovered and recrystallized easily during the tempering process. Thus, after tempering at 660 °C, the bainite ferrite grains became fine. In the following process of tempering, the M-A constituent is distributed as two parts; one part of the M-A constituent in the HAZ is decomposed, while the other is converted from slender into little dispersed particles.

## 5. Conclusions

(1) A 13MnNiMoR thick steel plate was welded by submerged-arc welding, with welding flux SJ102 and wire H10Mn2NiMoA. The microstructure of the welding joint in the as-welded condition is acicular ferrite and fine bainite, and the toughness value is high.
(2) Granular bainite occurred after hot stamping at 980 °C, normalizing at 920 °C, and intercritical normalizing at 740 °C; M-A constituents lead to the low toughness of the weld joint. After tempering from 620 to 660 °C followed by intercritical normalizing at 740 °C, most M-A constituents are decomposed, and bainite-ferrite in the weld and HAZ are transferred into recovered and recrystallized microstructure and fine ferrite, respectively. Thus, the toughness is improved and becomes higher than that in as-welded.
(3) During normalization at 740 °C, the concentration of Mo in ferrite was high and low in the austenite. After the cooling process, the austenite was converted into angular bainite, which results in low tempering resistance. After tempering from 620–660 °C, the angular bainite becomes recovered and recrystallized; thus, the toughness is improved.

## Figures and Tables

**Table 1:** The main composition of base metal (mass fraction %) and mechanical properties.

Element	C	Mn	Si	Cr	Ni	Mo	Nb	Rm (MPa)	Akv (−20 °C)
13MnNiMoR	0.13	1.47	0.4	0.28	0.82	0.32	0.014	>570	50

**Table 2:** The main composition of the SJ102 welding flux (mass fraction%).

Welding Flux	SiO_2_ + TiO_2_	CaO + MgO	Al_2_O_3_ + MnO	CaF_2_	S	P
SJ102	19.7	33.93	24.83	20.89	0.029	0.029

**Table 3:** The main composition of the welding wire (mass fraction %).

Element	C	Mn	Si	Cr	Ni	Mo	Cu	Ti	S	P
H10Mn2NiMoA	0.084	1.79	0.28	0.1	0.82	0.62	0.12	0.071	0.004	0.005

**Table 4:** The submerged-arc welding parameters of the experiment.

Layer	Welding Wire (Electrode) Diameter φ (mm)	Welding Current (A)	Welding Voltage (V)	Welding Speed (cm × min^−1^)
Backing welding	4(E6017)	160~180	22~24	20
Cosmetic welding	4(H10Mn2NiMoA)	450~480	30~32	43~45
Rest of welding	4(H10Mn2NiMoA)	580~600	30~32	42~44

**Table 5:** M-A constituent size and density for 980 °C for 1 h, 920 °C for 1 h (normalizing), 740 °C for 1 h (intercritical normalizing).

Heat Treatment	M-A Size in Weld (μm)	M-A Size in HAZ (μm)	M-A Density in Weld	M-A Density in HAZ
980 °C hot stamping	6	4	11.43%	8.84%
920 °C normalizing	11	2.5	19.69%	20.52%
740 °C intercritical normalizing	4	2.2	22.67%	23.67%

**Table 6:** M-A constituent size and density for tempering at 620 °C, 640 °C and 660 °C.

Tempered Temperature	M-A Size in Weld (μm)	M-A Size in HAZ (μm)	M-A Density in Weld	M-A Density in HAZ
620 °C	1.7	2.0	1.89%	2.41%
640 °C	1.6	1.8	1.21%	2.13%
660 °C	1.3	1.2	0.83%	0.43%

**Table 7:** The impact toughness of weld and HAZ in as-welded, hot stamping, normalization, intercritical normalization, tempered at 620 °C, 640 °C, and 660 °C, respectively.

Scheme	Position	Impact Energy (J)			Average (J)	Deviation (100%)
As-welded	Weld	60	59	49	56	8.9
As-welded	HAZ	94	110	126	110	11.9
Hot stamping	Weld	9	12	9	10	14.1
Hot stamping	HAZ	11	10	13	11	11.3
normalizing	Weld	10	11	8	10	12.5
normalizing	HAZ	9	11	10	10	8.2
Intercritical normalizing	Weld	19	17	13	16	15.6
Intercritical normalizing	HAZ	15	14	19	16	13.5
5(740 °C + 620 °C)	Weld	46	46	62	51	14.8
5(740 °C + 620 °C)	HAZ	64	64	68	65	2.9
6(740 °C + 640 °C)	Weld	66	53	50	56	12.4
6(740 °C + 640 °C)	HAZ	126	143	128	132	5.7
7(740 °C + 660 °C)	Weld	89	105	114	103	10.0
7(740 °C + 660 °C)	HAZ	169	129	116	138	16.3

## Data Availability

The data is available on request from corresponding authors.
